# Factors associated with stroke survivors’ inconsistent uptake of physiotherapy interventions at Turton Community Health Centre, KwaZulu-Natal

**DOI:** 10.4102/sajp.v76i1.1475

**Published:** 2020-10-07

**Authors:** Ntombifuthi Mlambo, Khumbulani Hlongwana

**Affiliations:** 1 Discipline of Public Health Medicine, School of Nursing and Public Health, University of KwaZulu-Natal, Durban, South Africa

**Keywords:** non-adherence, stroke survivors, physiotherapy treatment, physiotherapy interventions, inconsistent uptake

## Abstract

**Background:**

Stroke is one of the major causes of physical disability worldwide. Whilst physiotherapy interventions are important for the recovery of stroke survivors, the uptake remains inconsistent and factors contributing to these inconsistencies are not well documented, especially in South Africa.

**Objectives:**

The overall objective was to determine the intrinsic and extrinsic factors associated with adult stroke survivors’ inconsistent uptake of physiotherapy interventions at Turton Community Health Centre, Ugu District, KwaZulu-Natal, South Africa.

**Methods:**

This was a cross-sectional study involving 50 stroke survivors who missed one or more of their physiotherapy appointments and 25 who attended all their appointments (comparison group) within a 2-year period. A researcher-administered semi-structured questionnaire was used to collect data, which was captured and analysed using SPSS v25. Results were summarised using descriptive statistics. Pearson’s chi-square test was used for bivariate analysis.

**Results:**

Only two intrinsic factors were significantly associated with the outcome variable, namely: believed in exercises recommended by physiotherapists (χ^2^ = 3.86, *p* = 0.049) and improvements noted from the start of recommended exercises (χ^2^ = 9.439, *p* = 0.007). Transportation, including hiring of private cars (74%) and being far away from the health facility (48%), were key extrinsic challenges affecting access to health facilities.

**Conclusion:**

Personal reasons and the difficulty in accessing health facilities were main factors affecting stroke survivors’ uptake of physiotherapy interventions.

**Clinical implications:**

Design of patient-tracking and family support systems may potentially improve the stroke survivors’ uptake of physiotherapy interventions.

## Introduction

Stroke is a major cause of morbidity and mortality worldwide (Ntamo, Buso & Longo-Mbenza 2013), yet evidence suggests that it could be better managed through the improved uptake of physiotherapy interventions, with a view to minimise its long-term effects on patients (Jordan et al. 2011; Ntamo et al. 2013; Olaleye & Suddick 2012). Similar to the experiences of other stroke survivors, globally (Asvat 2011; Ntamo et al. 2013; Ogwumike, Badaru & Adeniyi 2015; Olaleye & Suddick 2012), inconsistent uptake of physiotherapy interventions remains a challenge amongst adult stroke survivors at Turton Community Health Centre (CHC), as documented in the 2015/16 Physiotherapy Departmental monthly statistics (Department of Health 2015/2016). Turton CHC is situated in Umzumbe, which is one of the six municipalities under the Ugu district in KwaZulu-Natal, South Africa. The 2015/16 Physiotherapy Departmental statistics revealed that as high as 60% of the stroke survivors had inconsistent uptake of scheduled physiotherapy appointments (Department of Health 2015/2016), and this has a negative effect on, amongst others, the stroke survivor’s quality healthcare outcomes and healthcare costs (Jack et al. 2010). Inconsistent uptake of physiotherapy interventions has been linked to a number of intrinsic and extrinsic factors. Intrinsic and extrinsic factors are defined as the determinants of inconsistent uptake of physiotherapy interventions that are within the control (intrinsic) of the stroke survivor, including personal attitudes and those that are outside the control (extrinsic) of the stroke survivor, including health systems issues (Asvat 2011; Jack et al. 2010).

The literature has documented a number of intrinsic and extrinsic factors affecting the uptake of physiotherapy interventions in various settings. These intrinsic factors include perceived barriers associated with one’s daily routine or low self-efficacy (Jack et al. 2010), pain intensity (Medina-Mirapeix et al. 2009b), the patient’s perceptions and knowledge of the disease (Mazières et al. 2008), patients forgetting their scheduled appointments (Asvat 2011; Jin et al. 2008; Potamitis et al. 1994), low levels of education, low socio-economic status, anxiety and depression, lack of motivation and negative attitude to therapy (Conraads et al. 2012; Duncan et al. 2005; Govender & Mash 2009; Jin et al. 2008; Martin et al. 2005). On the other hand, extrinsic factors include poor relationship between the patient and prescriber, the patient’s difficulties in accessing transport (Govender & Mash 2009; Jin et al. 2008; Kolapo & Vento 2011), long distances to clinics (Asvat 2011) and long waiting times at health facilities (Jin et al. 2008). However, physiotherapy interventions are still considered the effective options for managing stroke (Jordan et al. 2011; Ntamo et al. 2013; Olaleye & Suddick 2012), especially when survivors receive emotional support from friends and family members (Jin et al. 2008). Whilst, from time to time, physiotherapists receive large case load of stroke survivors (Olaleye & Suddick 2012), these survivors are often lost to follow up, which compromises the potential health benefits of physiotherapy interventions. Therefore, local data on the intrinsic and extrinsic factors associated with inconsistent uptake of physiotherapy interventions will help in the design of appropriate interventions.

The physiotherapy protocol or guidelines recommend that assessment of survivor’s functional status be performed to provide appropriate treatment and issue a tailored home programme (Van Peppen et al. 2008), the latter being the advice and exercises to do at home for continuity of therapy (Van Peppen et al. 2007). The exercises are useful in improving and maintaining functionality of affected body parts, promote self-management and reduce impairment (Olaleye & Suddick 2012). Patients with various conditions, including stroke, are therefore given follow-up appointment dates for further treatment, until they are fully discharged from physiotherapy interventions. However, effective tools for monitoring the survivors’ adherence to home programmes are still needed. Currently, a good attendance rate at physiotherapy is being used as the key indicator of clinic-based uptake of physiotherapy interventions (Al-Eisa 2010), yet even the very attendance is often not achieved, let alone weak and/or absence of monitoring tools for home programmes.

In the context of our study, the stroke survivors who missed their scheduled appointments on one or more occasions were categorised as having inconsistent uptake of physiotherapy interventions, irrespective of whether or not they consistently implemented their home programmes. Outpatients’ physiotherapy sessions are intended to prevent deterioration of a condition, reduce further complications that may arise, promote accession of activities of daily living, reduce pain and restore physical functioning (Ntamo et al. 2013). Despite the documented benefits of improved uptake of physiotherapy interventions (Ntamo et al. 2013), the proportion of patients with inconsistent uptake of these interventions is concerning. Therefore, the aim of our study was to investigate the intrinsic and extrinsic factors associated with inconsistent uptake of physiotherapy care by adult stroke survivors at Turton CHC, Ugu District, KwaZulu-Natal, South Africa.

## Method

The study was conducted through quantitative methods using a cross-sectional study design. It involved adult stroke survivors who had inconsistent uptake of physiotherapy interventions at Turton CHC and some stroke survivors who attended all their scheduled physiotherapy appointments were recruited as a comparison group. The inclusion of the reference group was necessary for determining the factors that had associations with inconsistent uptake of physiotherapy interventions. Turton CHC is a 24-h public primary healthcare facility situated in Umzumbe, which is one of the six municipalities falling under Ugu district in KwaZulu-Natal province, South Africa. This facility serves a catchment population of 47 922 and supports seven local clinics.

The records from patients’ files were reviewed by the first author at the Turton CHC Administration Department to identify eligible participants for recruitment and to obtain patients’ contact details. She reviewed files from the second week of November 2017 to mid-December 2017 and data collection lasted for about 3 months (mid-December 2017 to mid-March 2018). Eligible adult stroke survivors who missed one or more of their scheduled physiotherapy appointments within the previous 2 years (2015/16) preceding the data collection period and who could communicate were included in our study. Excluded from our study were stroke survivors with cognitive deficiencies, unable to communicate, aged below 18 years and referred to physiotherapy with acute conditions. The review of files and the data collection process began after our study had obtained ethical clearance from the university. Permission to do so was also granted by the Turton CHC Facility Management. No patient file was moved from the Administration Department, but was reviewed within the department.

**FIGURE 1:**
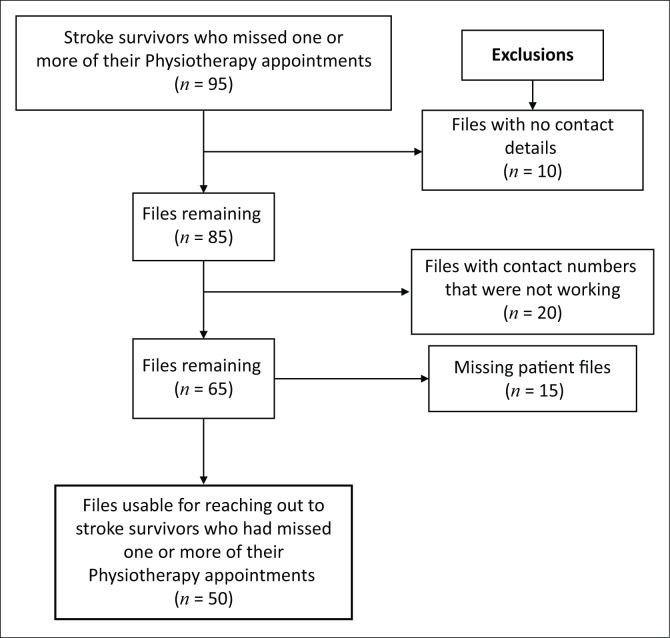
Identification of files for potentially eligible stroke survivors who missed one or more of their physiotherapy appointments.

Initial review showed that a total of 95 records were of stroke survivors who had been inconsistent in the uptake of physiotherapy interventions and had not attended all the scheduled physiotherapy appointments (Figure 1).

When files were individually scrutinised, 10 had no contact details, 20 files had contact numbers, but these numbers appeared to be faulty as they could not be used, and 15 patients on the register had missing files (Figure 1). The final number of files usable to contact stroke survivors who met the inclusion criteria was 50. Each stroke survivor was informed in detail about the purpose of the study, to avoid the study being misconstrued as punitive for their missed appointments, but that the goal of our study, was to learn more about the factors associated with their inconsistent uptake of physiotherapy interventions through missing one or more of their scheduled physiotherapy appointments (Asvat 2011). The inclusion and exclusion criteria were based on whether or not stroke survivors honoured their scheduled physiotherapy appointments, rather than adhering to the home programme.

The first author conducted home visits for the purposes of conducting the researcher-administered questionnaires, whereby all the selected stroke survivors, in addition to questionnaire administration, were also re-assessed and therapeutically managed, because they were defaulters anyway. This was possible because the first author is a practicing physiotherapist. The questionnaire used for data collection was revised several times by the second author, prior to being pretested on a group of colleagues to improve face and content validity. The semi-structured questionnaire covered stroke survivor-related factors, socio-economic factors and health service factors. The survivor-related factors included age, gender, level of education, cultural beliefs, depression and self-rated confidence, whereas socio-economic factors covered income, social grants and employment. Lastly, health service factors included satisfaction with healthcare, healthcare worker attitude and barriers to accessing care.

Twenty-five (25) stroke survivors who had honoured all their physiotherapy appointments were recruited as a comparison group. The recruitment of the 25 adherent stroke survivors was performed at the health facility, as they presented to the health centre for their appointments. Adult stroke survivors honouring their scheduled appointments and presenting at the physiotherapy department for their appointments during the data collection period were conveniently included in the study as a comparison group. The total number of stroke survivors participating in our study was 75, inclusive of survivors who were inconsistent in the uptake of physiotherapy interventions as they missed one or more of their scheduled physiotherapy appointments (*n* = 50) and stroke survivors who had attended all their scheduled physiotherapy appointment (*n* = 25). Data were collected through the researcher-administered semi-structured questionnaire, entered into and analysed using SPSS volume 25 statistical software.

Descriptive statistics, such as frequencies, percentages, means, median and standard deviation (SD), were used to summarise the data. A Pearson’s chi-square and Fischer’s exact test of association were used to determine factors associated with stroke survivors’ inconsistent uptake of physiotherapy interventions with a *p*-value < 0.05 deemed statistically significant.

### Ethical consideration

Our study obtained ethical approval from the University of KwaZulu-Natal’s Biomedical Research Ethics Committee (BREC: 486/17) on 06 Nov 2017, as well as the KwaZulu-Natal Provincial Department of Health Research Ethics Committee. Permission from Turton CHC was granted for conducting the study. Participants voluntarily signed the IsiZulu version of informed consent form. In the event where the potential participants were unable to sign, but consented to participate in our study, any family member of their choice was allowed to sign on their behalf.

## Results

### Demographic profile of the study participants

The mean ages and SD of the group with inconsistent uptake of physiotherapy interventions and the group with consistent uptake were 60 (SD = 14.748) and 56 (SD = 12.636) years, respectively.

More male participants were inconsistent in the uptake of physiotherapy treatment (56%), compared with their female counterparts (44%), with the number of consistent females being higher (60%) than that of their male (40%) counterparts (Table 1). A greater proportion of the consistent group (64%) had no formal education and 42% of the inconsistent group had primary education, with employment status being similar for both inconsistent and the consistent groups (Table 1). A number of participants were from the 49- to 58-year age group in both inconsistent participants (28%) and consistent participants (40%), respectively. The majority of participants were household heads in both inconsistent (66%) and consistent (76%) groups, so were the grant recipients, with a 94% and 96% in the inconsistent and the consistent groups, respectively (Table 1).

**TABLE 1:** Socio-demographic profile of inconsistent (*n* = 50) and consistent (*n* = 25) physiotherapy users.

Demographics	Inconsistent group		Consistent group	
	*n* = 50	%	*n* = 25	%
**Gender**				
Male	28	56	10	40
Female	22	44	15	60
**Age**				
19–28	1	2	0	0
29–38	2	4	2	8
39–48	7	14	4	16
49–58	14	28	10	40
59–68	11	22	5	20
69–78	11	22	2	8
79–88	4	8	2	8
**Highest education attained**				
No formal education	16	32	16	64
Primary	21	42	3	12
Secondary	12	24	6	24
Tertiary	1	2	0	0
**Employment status**				
Employed	0	0	0	0
Unemployed	3	6	1	4
Grant recipient	47	94	24	96
**Patient relationship with household head (Breadwinner)**				
Head	33	66	19	76
Spouse	4	8	2	8
Son or daughter	8	16	2	8
Grand child	0	0	0	0
Brother or sister	2	4	2	8
Nephew	1	2	0	0
Other	2	4	0	0

Inconsistent group: Mean age = 60 and standard deviation = 14.748.

Consistent group: Mean age = 56 and standard deviation = 12.636.

Participants were asked to share their households’ proximity to Turton CHC, with the results being comparable for both groups, except that no participant lived further than 60 km in the consistent group and also no participant lived 15 km or less away from the Turton CHC (Table 2).

**TABLE 2:** Distances between the stroke survivors’ residences and the Community Health Centre (*n* = 75).

Distance from TCHC	Inconsistent group		Consistent group	
	*n* = 50	%	*n* = 25	%
Less or equal 15 min (≤ 15 km)	1	2	0	0
15–29 min (16–30 km)	24	48	13	52
30–59 min (31–60 km)	19	38	12	48
1 hour–1 hour 29 min (61 km or more)	6	12	0	0

TCHC, Turton Community Health Centre

### Intrinsic and extrinsic factors associated with inconsistent uptake of physiotherapy interventions at Turton Community Health Centre

#### Intrinsic factors

Intrinsic factors considered for inconsistent uptake of physiotherapy interventions were as follows:

- self-reported knowledge about the stroke condition
- patient belief on the effectiveness of exercises recommended by physiotherapist
- whether or not improvements were noted by stroke survivors from the start of the recommended exercises
- stroke survivors’ recognition of the importance of implementing home programme exercises
- frequency of home programme exercises
- personal reasons for non-attendance of scheduled physiotherapy appointments. Notably, personal reasons, such as forgetfulness, are considered intrinsic, yet lack of transport money and family problems were classified as extrinsic factors.

Knowledge about the causes of stroke showed no association (*p* = 0.843) with whether or not patients would attend all their scheduled physiotherapy appointments, with 78% and 72% of inconsistent and consistent stroke survivors, respectively, reporting to have no knowledge of the causes of stroke (Table 3). Apart from the lack of association, most participants generally lacked knowledge about stroke (Table 3).

**TABLE 3:** Associations between a range of intrinsic and extrinsic variables and inconsistent uptake of physiotherapy interventions (overall *n* = 75).

Variable	Categories	Inconsistent group		Consistent group		*p*
		*n* = 50	%	*n* = 25	%	
**Intrinsic**						
Self-reported knowledge about the causes of stroke condition	Knowledgeable	5	10	3	12	0.843
	Not knowledgeable	39	78	18	72	
	Unsure	6	12	4	16	
Believed on exercises recommended by physiotherapist	Yes	43	86	25	100	0.049*
	No	7	14	0	0	
Improvements noted from the start of recommended exercises	Yes	28	56	19	76	0.007*
	No	22	44	6	24	
Consistent in the uptake of home programme exercises	Yes	46	92	25	100	0.294
	No	4	8	0	0	
Frequency of home programme exercises	Never	3	6	0	0	0.546
	At least once	47	94	25	100	
**Extrinsic**						
Mode of travelling to the clinic	By foot	1	2	0	0	0.736
	Private car hire	37	74	18	72	
	Public Transport	12	24	7	28	
Time taken to travel to the clinic	Less than minutes	1	2	0	0	0.266
	15–29 min	24	48	13	52	
	30–59 min	19	38	12	48	
	1 hour–1 hour 29 min	6	12	0	0	
Total amount spent on a trip to the clinic, using public or own transport	R0	1	2	0	0	0.261
	≤ R100	15	30	8	32	
	R101–R299	26	52	17	68	
	R300–R499	4	8	0	0	
	R500–R999	4	8	0	0	

Associations between recognising the importance of recommended exercise programme and consistent uptake of physiotherapy interventions were statistically significant (*p* = 0.049), with all (100%) consistent stroke survivors and 86% of their inconsistent counterparts, believing that exercises recommended by physiotherapist were effective (Table 3). The perceived improvements or lack thereof, from the initiation of the recommended exercises, were an important factor associated with stroke survivors deciding on whether or not to consistently attend all their scheduled physiotherapy appointments (*p* = 0.007). However, there was no statistically significant association between the uptake of home programme exercises in the stroke survivors who consistently attended all their scheduled physiotherapy appointments versus their inconsistent counterparts (*p* = 0.294), and in the frequency of home programme exercises (*p* = 0.546). For the inconsistent stroke survivors, family problems (42%) appeared to be the dominant challenges.

#### Extrinsic factors

Extrinsic factors considered for inconsistent uptake of physiotherapy interventions were as follows:

- mode of travelling to the clinic
- time taken to travel to the clinic
- total amount spent on a round trip to the clinic, using public or own transport.

Whilst a number of extrinsic factors appeared to have an association with inconsistent attendance to scheduled physiotherapy appointments, statistically significant associations could not be established. Seventy four per cent and 72% in the inconsistent and consistent groups, respectively, hired private cars to access the healthcare facility (Table 3). None of the stroke survivors who were consistent in attending all scheduled physiotherapy appointments reported to have travelled longer than 1 hour to the healthcare facility, compared with the 12% in their inconsistent counterparts. Most stroke survivors in both inconsistent (52%) and consistent (68%) groups spent between R100 and R299 to travel to the nearest clinic (Table 3) and the use of privately hired cars accounted for the greatest expenditure.

## Discussion

The objective of our study was to determine the intrinsic and extrinsic factors associated with inconsistent uptake of physiotherapy interventions, so that the results could be used to tailor targeted intervention strategies aimed at improving stroke survivors’ uptake of physiotherapy interventions. We found that perceived improvements or lack thereof for the stroke survivors’ from the initiation of recommended exercises, were associated with their decisions on whether or not to consistently attend all their scheduled physiotherapy appointments. On the other hand, the transport cost to the health facility was the key extrinsic factor contributing to stroke survivors’ inconsistent uptake of physiotherapy interventions. Therefore, ability of stroke survivors to recognise the importance of the exercise programme and transportation challenges remain the key intrinsic and extrinsic factors, respectively, affecting the survivors’ consistent uptake of physiotherapy interventions.

Similar to Jin et al. (2008), our study revealed that the age groups (49–58 years) and especially male participants were prone to being inconsistent in the uptake of physiotherapy interventions. These authors further recommended that programmes should take into account the age factor when designing interventions, despite the fact that only a few studies identified age as a contributor to the low uptake of physiotherapy interventions (Jack et al. 2010; Jin et al. 2008). Basically, evidence on the contribution of age to the low uptake of physiotherapy interventions is inconclusive (Jack et al. 2010; Jin et al. 2008). In our study, unemployed males, with primary level education and receiving disability grants (56%), had increased chances of being inconsistent in the uptake of physiotherapy interventions compared with their females (44%).

Jin et al. (2008) found inconsistent uptake amongst adult stroke survivors to be mostly unintentional, as elderly people rely on assistance from younger family members or healthcare providers to follow their prescribed therapies (Jin et al. 2008; Waari, Mutai & Gikunju 2018). Contrary to the findings of their study, Sluijs, Kok and Van Der Zee (1993) found that less educated patients had slightly increased adherence rate than that of highly educated patients.

Jin et al. (2008) have noted that results on the associations between gender and adherence to treatment in the few published studies present contradictory evidence. For example, some authors have found female patients to have better uptake of physiotherapy interventions, whilst the opposite is true in other studies, thereby making gender a weak predictor of non-adherence to treatment (Jin et al. 2008; Waari et al. 2018).

Family responsibilities also contributed towards low uptake of physiotherapy interventions as 66% of participants were breadwinners for their households, 54% were married and they had the responsibility of supporting their families through earning an income. About 94% depended on their limited government social grants (about R1780) for financial support, thus making it difficult to afford travel-related expenses to the healthcare facility to honour their scheduled appointments. A social grant, without complementary revenue, is inadequate to sustain patients’ daily living expenses for their households and still fully provide for regular physiotherapy visits. These results compare well with different studies conducted elsewhere in the world (Asvat 2011; Naidoo & Ennion 2019; Olaleye & Suddick 2012), in so far as the financial strain imposed by regular therapy visits, is concerned. Whilst gender, age, pain threshold and family responsibility appear to affect the stroke survivors’ uptake of physiotherapy interventions, they are based on weak evidence, which is inconclusive and at times contradictory.

Family challenges, including the unavailability of a family member to accompany the patient to scheduled appointments at the physiotherapy department, was one of the notable reasons for inconsistent uptake of physiotherapy interventions, as reported by 42% of the stroke survivors, who had missed one or more of their physiotherapy appointments. Stroke survivors often need the accompaniment of a family member or friend to appointments, given their limited mobility. This was indicative of the fact that the majority of stroke survivors experience challenges at home with their families, and these challenges hinder them from attending all their scheduled physiotherapy appointments. Several studies have also commented on the family challenges encountered by patients as they often depend on family members accompany them to the clinic (Jin et al. 2008; Kagee, Le Roux & Dick 2007; Medina-Mirapeix et al. 2009a, 2009b). However, these family members have other responsibilities of their own and may not always be readily available and/or even prepared to assist, at a time when the patient needs them. Perhaps, they themselves require emotional preparation and caregiving tips to provide better care to stroke patients, over and above being physically available to provide care.

It is therefore clear that stroke survivors’ uptake of physiotherapy interventions requires a partnership between the survivor and the family. Studies on the uptake of these interventions should equally consider the role of families during the care continuum.

Lack of knowledge about stroke and physiotherapy interventions in our study appeared to increase the risk of inconsistent uptake of physiotherapy interventions. However, the associations between the lack of knowledge and inconsistent uptake of physiotherapy interventions were not statistically significant. These findings were similar to the results of other studies, which investigated the association between demographic characteristics and compliancy of stroke survivors to home programme exercises (Adeniyi & Zandam 2012; Naidoo & Ennion 2019). Continuous rehabilitation, using home programmes and physiotherapy sessions is known to improve mobility, functionality and prevention of further complications. The inaccessibility to healthcare services, including rehabilitation treatments by people with disabilities in South Africa, is well documented (Naidoo & Ennion 2019). In our study, financial constraints and proximity to health facilities deterred stroke survivors from attending rehabilitation sessions, a phenomenon that is seen in other studies (Jack et al. 2010; Kagee et al. 2007; Naidoo & Ennion 2019; Ntamo et al. 2013; Ogwumike et al. 2015). Most of the participants (74%) hired private transport to commute to the health facility, costing an average amount of R300 for a round trip. This amount is too much for people who live below the poverty margin. Public transport in South Africa, such as taxis, operate within the community during the day and are considered cheaper than hiring a private car. However, a key challenge with public transport is the pick-up and drop-off points. These points are often distant from patients’ houses, yet these patients require transport from their houses to the taxi or bus stop, as well, given their limited mobility. The link between transport challenges and non-adherence to treatment has also been established in other studies (Asvat 2011; Jack et al. 2010; Kagee et al. 2007; Naidoo & Ennion 2019). In our study, the association between non-adherence and travel to the healthcare facility, including the distance, was not statistically significant. Whilst poor accessibility to health facilities appears to negatively affect the stroke survivors’ uptake of physiotherapy interventions, factors deterring the stroke survivors’ uptake of physiotherapy interventions are complex and multifaceted.

More than half (58%) of the stroke survivors participating in our study were not consistently implementing their home programmes, because of the survivors’ misconstrued delay in achieving positive health outcomes as a programme failure. If patients do not see results immediately after therapeutic programmes commence, they often lose faith in the validity of prescribed treatment (Al-Eisa 2010). Low uptake of interventions can be attributed to factors, such as, lack of visual aids being provided to remind patients on their home programme exercises (Kagee 2004). Time and effort needed to complete the exercises may also be a contributing factor, as well as fear of injury in the absence of a therapist, whilst commencing with the home programme (Ogwumike et al. 2015; Olaleye & Suddick 2012). The relationship between unsupervised home programme exercises and low uptake is similar to the study conducted by Adeniyi et al. in Kano, Nigeria (Adeniyi & Zandam 2012). Stroke survivors are health outcome-driven and may not exercise patience until results are yielded. Perhaps, this talks to the missed opportunities by healthcare practitioners in utilising various resources, such as behaviour change models, to enhance stroke survivors’ uptake of physiotherapy interventions (Basset 2015; Keogh et al. 2015; Willet et al. 2017).

To the best of the authors’ knowledge, this is the first study investigating factors associated with inconsistent uptake of physiotherapy interventions amongst adult stroke survivors in KwaZulu-Natal namely the Ugu district, in particular. The findings may be useful in developing more appropriate interventions to improve the uptake of physiotherapy.

The study was conducted in a rural setting with poor road infrastructure, thereby prolonging the data collection process, especially during inclement weather conditions. Self-reporting made the study vulnerable to social desirability and recall bias.

Our study results cannot be generalised to all stroke survivors in Umzumbe Municipality for two reasons namely: it excluded patients who attended private physiotherapists and the sample size was too small to draw conclusions that can be generalised.

### Implications of the study

Our results provide a better understanding of the key factors associated with stroke survivors’ inconsistent uptake of physiotherapy interventions in Umzumbe Municipality. These results could be used for targeted interventions aimed at improving stroke survivors’ uptake of physiotherapy interventions. We suggest the need for a re-think into establishing physiotherapy outreach teams for stroke survivors, given the mobility challenges they face, which, in turn, hinder their attendance to physiotherapy appointments. This is further motivated by the first author’s observation of the patients’ appreciation of being provided with assessment in the comfort of their homes, although the data collection tools had not accommodated for these observational data.

## Conclusion

The results of our study revealed that challenges affecting the uptake of physiotherapy interventions by stroke survivors are threefold, and these include personal (intrinsic) support from the family and health systems (extrinsic) factors. Intrinsic factors should be addressed through targeted interventions that are goal-driven and collaboratively set by the stroke survivor and the physiotherapist. On the same score, survivors’ expectations need to be addressed, so that they are realistic about the expected therapeutic outcomes of the physiotherapy interventions. Finally, the design of patient-tracking and family support systems may potentially improve survivors’ uptake of physiotherapy interventions.
