# Erratum: Factors associated with stroke survivors’ inconsistent uptake of physiotherapy interventions at Turton Community Health Centre, KwaZulu-Natal

**DOI:** 10.4102/sajp.v77i1.1612

**Published:** 2021-11-19

**Authors:** Ntombifuthi Mlambo, Boikhutso Tlou, Khumbulani Hlongwana

**Affiliations:** 1 Discipline of Public Health Medicine, School of Nursing and Public Health, University of KwaZulu-Natal, Durban, South Africa

In the version of this article initially published, Mlambo, N. & Hlongwana, K., 2020, ‘Factors associated with stroke survivors’ inconsistent uptake of physiotherapy interventions at Turton Community Health Centre, KwaZulu-Natal’, *South African Journal of Physiotherapy* 76(1), a1475. https://doi.org/10.4102/sajp.v76i1.1475, the name of the second author has unintentionally been omitted in the ‘Authors’ and ‘How to cite this article’ sections. The second author’s name should be included as Boikhutso Tlou.

The ‘Authors’, ‘How to cite this article’ and ‘Authors’ contributions’ sections are now corrected as:

**Authors:**

Ntombifuthi Mlambo^1^ https://orcid.org/0000-0003-4559-5143

Boikhutso Tlou^1^ https://orcid.org/0000-0003-1732-1903

Khumbulani Hlongwana^1^ https://orcid.org/0000-0002-1777-0402

**How to cite this article:**

Mlambo, N., Tlou, B. & Hlongwana, K., 2021, ‘Erratum: Factors associated with stroke survivors’ inconsistent uptake of physiotherapy interventions at Turton Community Health Centre, KwaZulu-Natal’, *South African Journal of Physiotherapy* 77(1), a1612. https://doi.org/10.4102/sajp.v77i1.1612

**Authors’ contributions:**

N.M. conceptualised the study, designed the study, collected and analysed data and wrote up the manuscript. K.H. guided the conceptualisation of the study, study design and the write-up. N.M. and K.H. participated in drafting this manuscript and approved the submission. B.T. provided scientific and statistical inputs and insights to the article.

This correction does not alter the study’s findings of significance or overall interpretation of the study results. The publisher apologises for any inconvenience caused.

